# Effect of Tungsten Inert Gas Remelting on Microstructure and Corrosion Resistance of Q450NQR1 High-Strength Weathering Steel-Welded Joints

**DOI:** 10.3390/ma17051198

**Published:** 2024-03-04

**Authors:** Xuemei Li, Yang Liu, Rui Guo, Zicheng Li, Qingming Hu, Meng Liu, Lei Zhu, Xiangxia Kong

**Affiliations:** 1 School of Mechanical and Electronic Engineering, Qiqihar University, Qiqihar 161006, China; 15853612066@163.com (Y.L.); 15326646620@163.com (R.G.); lzc0802@126.com (Z.L.); 02510@qqhru.edu.cn (Q.H.); liumeng5201@126.com (M.L.); 02648@qqhru.edu.cn (L.Z.); 2 CRRC Qiqihar Rolling Stock Co., Ltd., Qiqihar 161002, China; 3 The Engineering Technology Research Center for Precision Manufacturing Equipment and Industrial Perception of Heilongjiang Province, Qiqihar 161006, China; 4 School of Material Science and Engineering, Harbin University of Science and Technology, Harbin 150080, China; 5 Department of Material Engineering, North China Institute of Aerospace Engineering, Langfang 065000, China; kongxx@nciae.edu.cn

**Keywords:** weathering steel, TIG remelting, corrosion resistance, microstructure

## Abstract

In this paper, the corrosion environment of a railway coal truck was simulated with 1.0%H_2_SO_4_ + 3%NaCl solution. The effect of weld toe Tungsten Inert Gas (TIG) remelting on the microstructure and corrosion resistance of welded joints of Q450NQR1 high-strength weathering steel was studied. The results show that the weld toe melts to form a remelting area after TIG remelting. After TIG remelting, the weld geometry was improved, and the stress concentration factor decreased from 1.17 to 1.06 at the weld toe, a decrease of 9.4%. TIG remelting refines the microstructure of the weld toe and improves the corrosion resistance of the welded joint. The surface of the TIG-remelted sample is uniformly corroded with no “deep and narrow” pits after the removal of corrosion products. The weight loss rate and corrosion rate of remelted welds are lower than those of unremelted welds. The structure of corrosion products is loose at the initial stage of corrosion, and the corrosion products are transformed into Fe_3_O_4_ and Fe_2_O_3_ protective rust layers with a dense structure after 480 h of corrosion. With the extension of corrosion time, the tensile strength and percentage elongation of the specimen decreased linearly. The decreasing rates of tensile strength of remelted and unremelted specimens were 0.09 and 0.11, respectively, and the decreasing rates of elongation after fracture were 0.0061 and 0.0076, respectively.

## 1. Introduction

Q450NQR1 high-strength weathering steel is the main type of material used in the manufacture of railway freight wagons because it has high mechanical strength, good atmospheric corrosion resistance, and good weldability, which meet the requirements of high-speed and heavy-duty use. A welded structure is the main connection mode of railway freight wagon steel structures because of its high flexibility and high strength. The welding local heating process leads to uneven composition, microstructure, properties, and large residual stress at the welding joint [1,2,3]. Therefore, the mechanical properties and corrosion resistance of welded joints are relatively weak [4,5,6].

A general purpose open top wagon is the main means of coal transportation. The working environment of the coal wagon is an acidic environment containing Cl^−^, in which Cl^−^ mainly comes from coal leaching products and antifreeze, and the acid environment is mainly acid rain and sulfate in coal dissolved in water to form HSO_4_^−^ [7,8]. Wagon steel structure welded joints have uneven microstructure and residual stress, so its corrosion resistance is lower than that of base metal [9,10]. Therefore, it is of great significance to study the welded structures corrosion behavior of railway freight wagons in acidic environments containing Cl^−^ and SO_4_^2−^.

Tungsten inert gas (TIG) remelting is a technological process in which the already welded joint is reheated and remelted using the TIG welding method without the addition of filler wire. This process smooths the weld toe area and eliminates possible solidification defects that formed during welding, by remelting a slightly expanded area of it. Stress concentration at the weld toe is reduced, and the microstructure and mechanical properties of the welded joint are improved. The research results of Zhu et al. show that the residual stress on the surface of the weld toe can be transformed into biaxial compressive stress by applying a cold source when the remelting zone of TIG is reheated to above 450 °C [11]. Abboud et al. found that the cooling rate of nodular cast iron surface is fast during TIG remelting, the microstructure of nodular cast iron is refined, and graphite is transformed into eutectic carbide, which enhances the corrosion resistance of nodular cast iron surface [12]. Zhu et al. found that the microhardness of the remelted layer increased, the impact toughness decreased and the wear resistance improved after TIG remelting treatment on the surface of 4Cr5MoSiV1 steel [13]. Al-Karawi found that the fatigue life of TIG-HFMI combined treatment is always longer than that of any single treatment [14]. The study by Mitelea et al. shows that the surface hardness increases and the cavitation erosion resistance improves after TIG remelting on the surface of gray cast iron [15].

At present, researchers should pay attention to the effect of TIG remelting on microstructure and mechanical properties, but the corrosion resistance of welded joints after TIG remelting has not been reported. In this work, Q450NQR1 steel was welded using the metal active gas (MAG) process, and then the MAG welding weld toe was remelted using the TIG process. The microstructure changes in welds after TIG remelting were studied. The micro-morphology of the corrosion surface and the formation process of corrosion products were studied, and the effect of TIG remelting on the corrosion resistance of welded joints was analyzed.

## 2. Materials and Methods

The experimental base material is Q450NQR1 high-strength weathering steel, and the test plate size is 350 mm × 150 mm × 5 mm with an assembly gap of 2 mm. The welding wire used is HTW-55 with a diameter of 1.2 mm. The chemical compositions of Q450NQR1 high-strength weathering steel and HTW-55 are shown in Table 1.

Double-sided welding of the test plate using MAG welding is used to obtain MAG weld. TIG remelting of the weld toe on one side of the test plate after MAG welding. The specific welding process parameters for MAG and TIG remelting are shown in Table 2. To ensure the weld quality, a 100% ultrasonic inspection of the weld is carried out after welding. MAG specimens and TIG remelting specimens were obtained by wire cutting after welding. The samples were taken with the weld center as the axis of symmetry, with dimensions of 50 mm × 15 mm × 5 mm.

The corroded specimen surface needs to be polished to remove the surface oxide film before corrosion. The specimen should be thoroughly cleaned with anhydrous ethanol and weighed after drying. The corrosion experiment is conducted according to ASTM G31-21 [16]. During the corrosion experiment, the 1.0% H_2_SO_4_+3% NaCl mixture was used to simulate the corrosive environment of the coal wagon. The corrosion period of the specimens is 240 h, 360 h, 480 h, and 600 h, with three parallel specimens used for each corrosion period. To ensure the stability of the corrosive ability of the corrosion solution, a new corrosion solution is added to the corrosion solution every three days during the corrosion process to maintain a constant pH value. The samples were treated with a 12% hydrochloric acid solution to remove the corrosion products after corrosion. The specimens, after rust removal, were thoroughly cleaned using anhydrous ethanol and then weighed after drying. The corrosion weight loss rate (*η_w_*) and corrosion rate (*V*) of the sample are calculated by the following equation [17].

(1) $$\eta_{w}=\frac{W_{0}-W_{1}}{W_{0}}\cdot 100\%$$

(2) $$V=\frac{W_{0}-W_{1}}{St}$$

where *V* is the corrosion rate (g), W_0_ is the original weight of the sample (g), *W*_1_ is the weight of the sample after removal of corrosion products (g), S is the surface area of the sample exposed to corrosion solution (m^2^), t is the experimental period (h).

**Figure 1:**
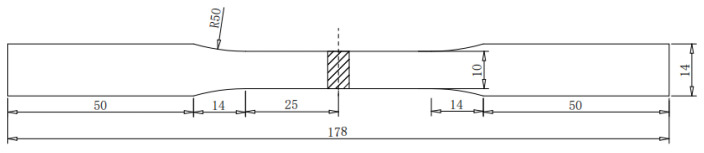
Schematic diagram of tensile specimen.

The microstructure of the TIG remelting weld joint was obtained using a metallographic microscope, while the micro-morphology of corrosion products and the micro-morphology of the specimen surface after corrosion product removal were obtained using scanning electron microscopy (SEM). The sample needs to be mechanically ground and polished before metallographic observation, followed by etching the sample surface with the 4% nitric acid solution. The tensile tests use an electromechanical universal material tensile testing machine. The non-standard tensile specimen shown in Figure 1 is used to test the tensile properties, and the residual height of the tensile specimen weld is not removed.

## 3. Results and Discussion

### 3.1. Weld Toe Stress Concentration

**Figure 2:**
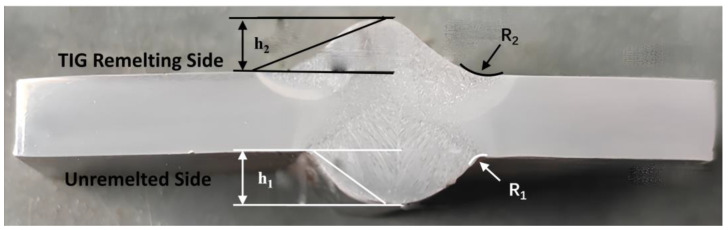
The cross-sectional morphology of the test specimen after TIG remelting.

The macroscopic morphology of the welded joint after TIG remelting is shown in Figure 2. The weld reinforcement h_1_, transition angle θ_1_ and curvature radius r_1_ of the weld seam before TIG remelting, and weld reinforcement h_2_, transition angle θ_2_, and curvature radius r_2_ of the weld seam after TIG remelting are shown in Table 3. After TIG remelting, weld reinforcement and the transition angle significantly decrease, the curvature radius increases, and the transition between the weld and the base metal becomes smoother.

The weld reinforcement and transition angle of a weld joint determine the stress concentration factor (*K_t_*) of the weld. *K_t_* can be obtained from the following equation [18]:

(3) $$k_{t}=1+\frac{1-exp\left[-0.9\sqrt{\frac{B}{h}}\left(\pi -\theta \right)\right]}{1-exp\left[-0.9\sqrt{\frac{B}{h}}\cdot \frac{\pi }{2}\right]}\cdot {\left[\frac{b-2}{2.8B}\cdot \frac{h}{R}\right]}^{0.65}$$

where *h* represents weld reinforcement (mm); *b* represents half of the specimen thickness (mm); *B* = *h* + *b*; *θ* represents transition angle (rad); R represents radius of curvature (mm).

The stress concentration factors *K_t_*_1_ and *K_t_*_2_ for the weld joint before and after remelting were calculated to be 1.17 and 1.06, respectively. Calculations resulted in a decrease in the value of the stress concentration coefficient by 9.4% after performing the TIG remelting treatment, which proves that the geometric shape of the weld has been improved by this procedure and a more favorable behavior in terms of the mechanical requirements is expected during exploitation.

### 3.2. Effect of TIG Remelting on Microstructure of Welded Joint

**Figure 3:**
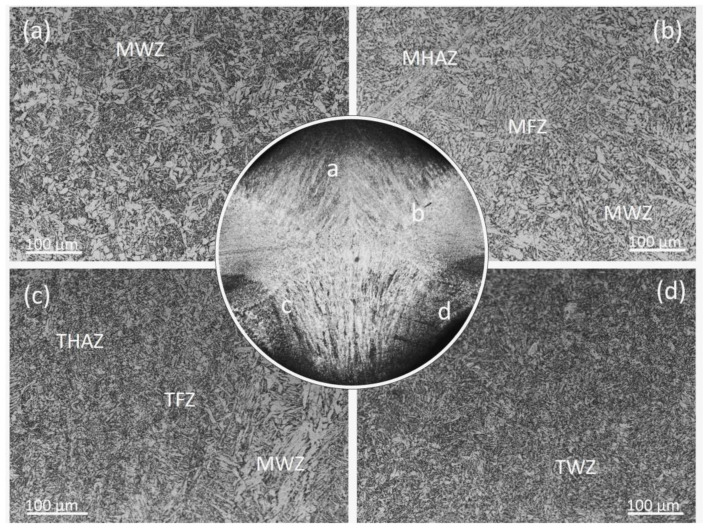
Microstructure of welded joints between unremelted and TIG remelting specimens (**a**) MWZ (**b**) MFZ (**c**) TWZ (**d**) TFZ.

Figure 3 shows the microstructure of the MAG weld zone (MWZ), MAG fusion zone (MFZ), MAG heat-affected zone (MHAZ), TIG remelting weld zone (TWZ), TIG remelting heat-affected zone (THAZ), and the TIG remelting fusion zone (TFZ) near the MAG weld side. Figure 3a shows that the microstructure of the MWZ consists of primary ferrite, acicular ferrite, and a small amount of pearlite. As shown in Figure 3b, the MWZ near the MAG fusion line is mainly columnar primary ferrite and pearlite, and the MHAZ near the MAG fusion line is coarse blocky ferrite and pearlite. After TIG remelting treatment, the weld toe is heated and remelted to form TWZ and THAZ. Figure 3c shows the microstructure at the junction of the TIG remelting weld and unremelted weld. It is obvious that the columnar grains and coarse ferrite in the MHAZ are refined after TIG remelting. The ferrite in the weld after TIG remelting is mainly in the form of acicular ferrite, as shown in Figure 3d. The reason for this is that the heat input during TIG remelting is small, resulting in a fast cooling rate of the weld joint and an increased undercooling degree of the austenite [19]. The increase in undercooling of austenite will enhance the nucleation rate of acicular ferrite within the austenite, thereby reducing the available space for free growth of acicular ferrite. Therefore, the grains in the TWZ and THAZ are refined.

### 3.3. Effect of TIG Remelting on Corrosion Resistance of Welded Joint

#### 3.3.1. Macroscopic Morphology Analysis of Corrosion

**Figure 4:**
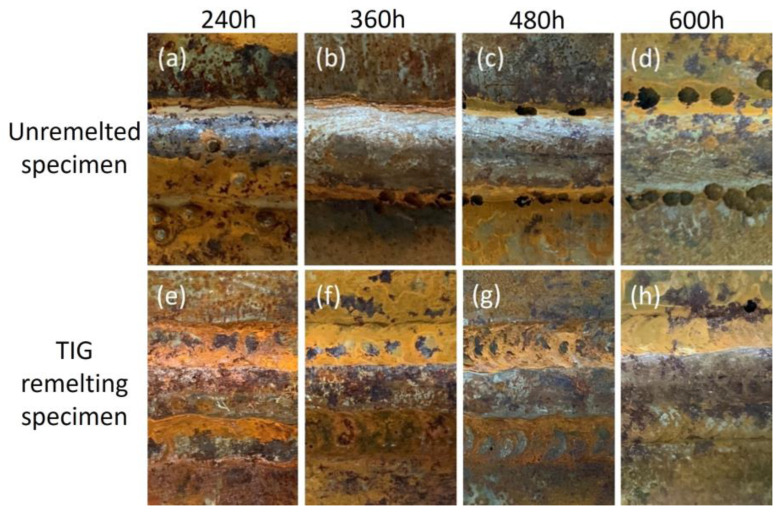
Macroscopic morphology of the corroded surface of welded joints at different corrosion periods. (**a**) unremelted 240 h, (**b**) unremelted 360 h, (**c**) unremelted 480 h, (**d**) unremelted 600 h, (**e**) TIG remelting 240 h, (**f**) TIG remelting 360 h, (**g**) TIG remelting 480 h, (**h**) TIG remelting 600 h.

Figure 4 shows the welded joints’ corrosion surface macroscopic morphology. The test piece that has not been remelted has holes in the weld due to local corrosion at the weld toe. As the corrosion time increases, the size and quantity of the holes increase, as shown in Figure 4a–d. The main form of corrosion observed on weld toes during the TIG remelting joints corrosion process is uniform corrosion. No holes are observed even after 600 h of corrosion, as shown in Figure 4h. The reason for this phenomenon is that TIG remelting refines the microstructure at the weld toe, reduces defects and residual stress at the weld toe, and improves the corrosion resistance of the weld toe.

#### 3.3.2. Micro-Morphology Analysis of Corrosion

**Figure 5:**
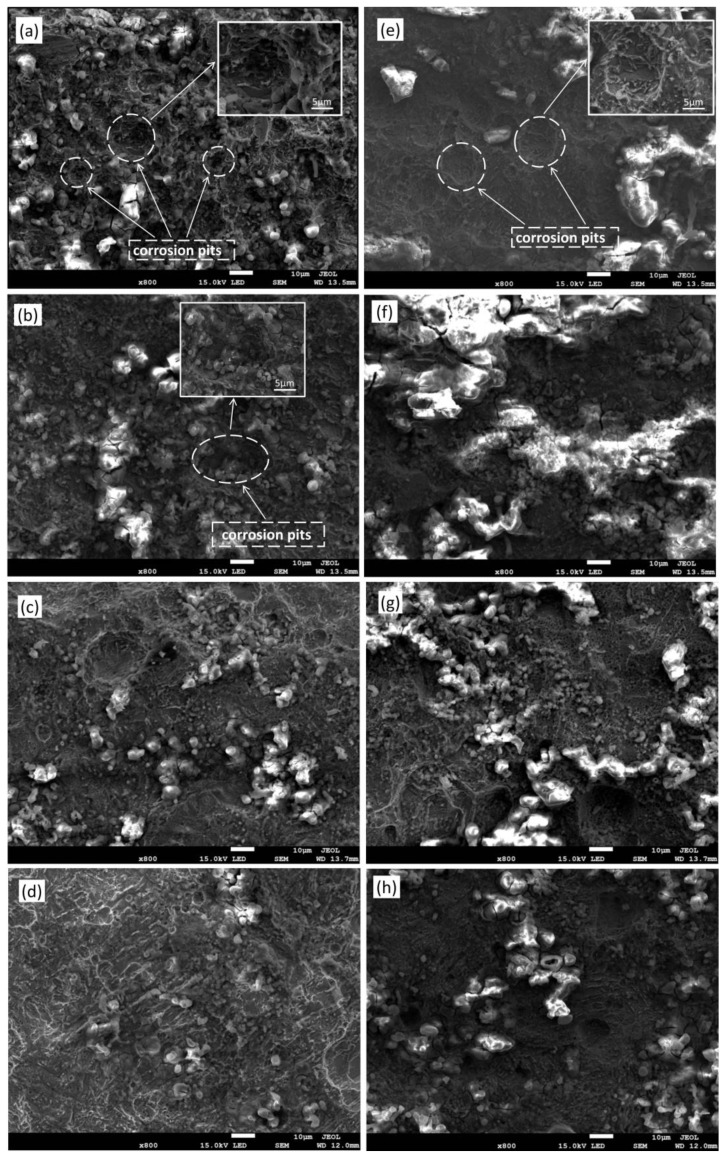
The surface morphology of the heat-affected zone after removal of corrosion products from TIG remelting and unremelted specimens. (**a**) unremelted 240 h, (**b**) unremelted 360 h, (**c**) unremelted 480 h, (**d**) unremelted 600 h, (**e**) TIG remelting 240 h, (**f**) TIG remelting 360 h, (**g**) TIG remelting 480 h, (**h**) TIG remelting 600 h.

Figure 5 shows the surface morphology of the heat-affected zone after the removal of corrosion products from TIG remelting and unremelted specimens. When the corrosion time reached 240 h, the unremelted sample weld surface was severely damaged, with many pits formed, as shown in Figure 5a. Some of the erosion pits have relatively large dimensions and depths. The PH value of the corrosion solution inside the pit increases as corrosion progresses, which leads to a decrease in the corrosion rate at the bottom of the pit. Therefore, the growth rate in the depth of the pit is smaller than the growth rate in the diameter of the pit, and small-diameter pits merge and fuse into larger pits with shallower depths (Figure 5b). When the corrosion time reaches 480 h, only a small amount of shallow corrosion pits remains on the weld surface, as shown in Figure 5c. When the corrosion time reaches 600 h, the planarization trend of corrosion pits on the surface of the unremelted weld is obvious, but there are still some corrosion pits with regular shapes. When the corrosion time reaches 240 h, the corrosion surface of the TIG remelting weld is primarily characterized by uniform corrosion (Figure 5e). There are a few shallow corrosion pits present on the test sample surface. As the corrosion continues, there is no obvious change on the weld surface after TIG remelting, and the shallow corrosion pits gradually expand smoothly, connecting with adjacent corrosion pits and evolving into irregular corrosion pits, as shown in Figure 5f,g. After 600 h of corrosion, the TIG remelting specimen reached a relatively stable corrosion process, with no apparent corrosion pits observed (Figure 5h).

The reason for the difference in surface morphology between the TIG remelting and unremelted specimens after removing corrosion products is that the unremelted samples contain a large number of columnar crystals and have a more uneven microstructure within the weld seam. The corrosion pits exhibit a “deep and narrow” morphology [20]. The TIG remelting process induces grain refinement at the weld toe, resulting in lateral dissolution of the material rather than deep dissolution, effectively suppressing the formation of “deep and narrow” corrosion pits.

### 3.4. Corrosion Weight Loss Rate and Corrosion Rate

Table 4 presents the weight loss rate and corrosion rate of TIG remelting and unremelted specimens after different corrosion periods. The data were processed using the specialized software Origin (v2018) and the corresponding evolution equation resulted.

**Figure 6:**
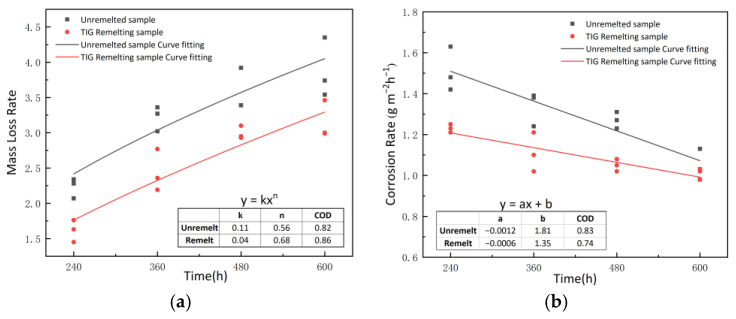
Average weight loss rate (**a**) and corrosion rate (**b**) of unremelted and TIG remelting samples under different corrosion periods.

Figure 6 shows the fitting relationship between corrosion time and weight loss rate, corrosion rate. Figure 6a shows the relationship between the corrosion time and the weight loss rate of the specimen. Throughout the corrosion process, the weight loss rate of the TIG remelting specimens is lower than that of the unremelted specimens. The weight loss rate of TIG remelting and unremelted specimens exhibits the same trend, with an increasing trend in weight loss rate as the corrosion time prolongs. The relationship between weight loss rate and corrosion time follows a power function [21,22]. The values of A, n, and COD obtained by fitting with a power function are presented in the table shown in Figure 6a.

Figure 6b shows the relationship between corrosion rate and corrosion time. The fitted values of a, b, and COD are presented in the table in Figure 6b. From the figure, it can be observed that the corrosion rate gradually decreases as corrosion progresses. Within any given corrosion period, the corrosion rate of TIG remelting specimens is lower than that of unremelted specimens. The decrease rate of corrosion rate for TIG-remelted specimens is lower than that for unremelted specimens. After a corrosion time of 600 h, the corrosion rates of both specimens become similar.

The reason for the above phenomenon is that the thickness and density of the rust layer on the steel substrate surface continue to increase during the corrosion process. The rust layer plays a role in protecting the substrate and increasing its corrosion resistance. Therefore, the growth rate of the weight loss rate and the corrosion rate of the specimen both decrease as the corrosion time prolongs. The unremelted sample exhibits inhomogeneity in the microstructure at the weld toe and significant residual stresses after welding [23,24]. Localized corrosion is more severe in the early stage of corrosion in the MWZ and MHAZ, and a large number of holes are generated at the weld toe during the corrosion process. A remelted weld is formed at the weld toe after TIG remelting, which reduces the number of defects at the weld toe. The rapid cooling rate during TIG remelting refines the grains in the MWZ and MHAZ, eliminating coarse columnar grains and forming fine acicular ferrite grains. Therefore, the corrosion weight loss rate and corrosion rate of TIG remelting specimens during corrosion are lower than those of unremelted welds. When the corrosion time reaches 480 h, the specimen corrosion product becomes dense, and the influence of microstructure and stress state on the corrosion process decreases. The corrosion rate of the TIG remelting specimen is similar to that of the unremelted specimen until the corrosion time reaches 600 h.

### 3.5. Analysis of Corrosion Products

**Figure 7:**
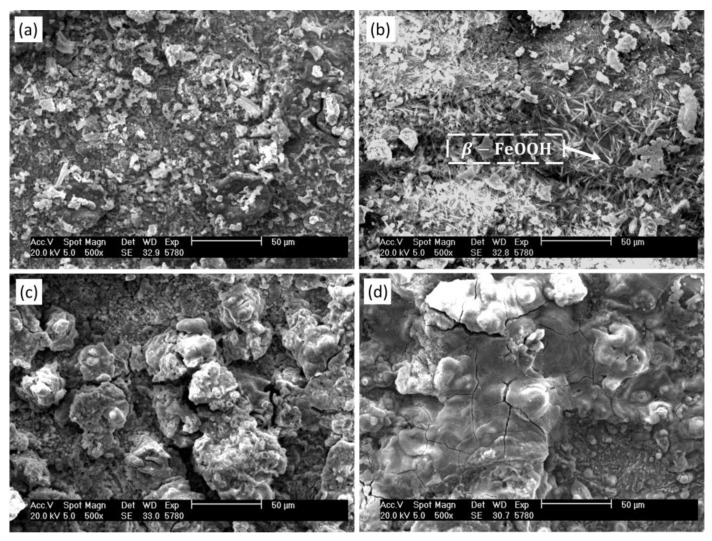
The corrosion products microstructure on the TIG remelting specimens surface. (**a**) 240 h, (**b**) 360 h, (**c**) 480 h, (**d**) 600 h.

For the Q450NQR1 welded joint, the corrosion products of the unremelted specimen and the TIG remelting specimen at different corrosion stages are the same. Figure 7 shows the microstructure of the surface corrosion products of TIG remelting specimens at different corrosion times. In the initial stage of corrosion, the structure of corrosion products is loose, making it easy for the corrosive medium to penetrate through the gaps of the corrosion products and come into contact with the substrate, thus further promoting the corrosion process, as shown in Figure 7a. When the corrosion time is 360 h, the corrosion products on the surface of the sample form needle-like crystals, and existing research results indicate that these needle-like crystals are β-FeOOH, as shown in Figure 7b. After the formation of the β-FeOOH needle-like structure, there are corrosive gaps present on the sample surface, facilitating further corrosion of the substrate. As corrosion progresses, the corrosion products gradually increase, and the integrity of the rust layer grows. When the corrosion time reaches 480 h, plate-like Fe_2_O_3_ and Fe_3_O_4_ are formed on the surface of the specimen, as shown in Figure 7c. The corrosion products gradually accumulate and eventually form a protective rust layer with stable performance and dense structure, as shown in Figure 7d.

During the corrosion process of Q450NQR1 weathering steel, electrochemical corrosion occurs first on the metal surface. The anodic reaction is as follows:

(4) $$\mathrm{Fe}\to {\mathrm{Fe}}^{2+}+2e^{-}$$

The cathodic reaction is mainly the hydrogen evolution reaction.

(5) $$2H^{+}+2e^{-}\to H_{2}$$

Some free Fe^2+^ and Cl^−^ ions can combine to form ferrous chloride.

(6) $${\;\mathrm{Fe}}^{2+}+2{\mathrm{Cl}}^{-}\to {\mathrm{FeCl}}_{2}$$

In high Cl^−^ concentration and low pH environments, the formation of hydroxychloride of iron [β-Fe_2_(OH)_3_Cl] and GRI [Fe_3_^II^Fe^III^(OH)_8_Cl^−^nH_2_O] can be promoted [25,26]. They serve as intermediate products, which are subsequently transformed into β-FeOOH, as shown in the following equation:

(7) $${\mathrm{FeCl}}_{2}\to \beta -{\mathrm{Fe}}_{2}{\left(\mathrm{OH}\right)}_{3}\mathrm{Cl}\to \mathrm{GRI}\left({\mathrm{Cl}}^{-}\right)\;\to \beta -\mathrm{FeOOH}$$

As corrosion progresses, the pH value of the solution increases, and the remaining Fe^2+^ continues to convert to Fe_2_ (SO_4_)_3_·8H_2_O and Fe_2_O_3_. The reaction equation is as follows:

(8) $$12{\mathrm{Fe}}^{2+}+12{\mathrm{SO}}_{4}^{2-}+3O_{2}+38H_{2}o\to {\mathrm{Fe}}_{2}{\left({\mathrm{SO}}_{4}\right)}_{3}\cdot 8H_{2}O+4\mathrm{Fe}{\left(\mathrm{OH}\right)}_{3}$$

(9) $$2\mathrm{Fe}{\left(\mathrm{OH}\right)}_{3}\to {\mathrm{Fe}}_{2}O_{3}\cdot H_{2}O+2H_{2}O$$

The FeOOH with poor stability is further transformed into more stable products, such as Fe_2_O_3_ and Fe_3_O_4_.

(10) $$2\mathrm{FeOOH}\to \gamma -{\mathrm{Fe}}_{2}O_{3}+4H_{2}O$$

(11) $$3\mathrm{FeOOH}+\frac{1}{2}O_{2}+3H^{+}\to {\mathrm{Fe}}_{3\;}O_{4}+3H_{2}O$$

### 3.6. Analysis of Tensile Performance and Mechanical Degradation of Corroded Specimens

Table 5 presents the tensile strength and percentage elongation after fracture of TIG remelting and unremelted specimens after different corrosion periods. Fit the data using the Origin graphing software and provide the corresponding fitting equation.

**Figure 8:**
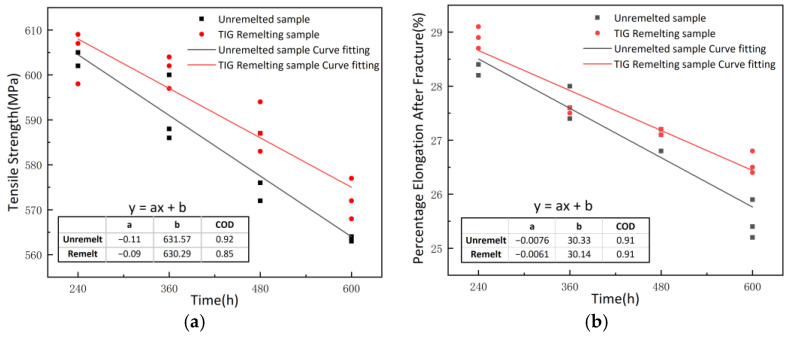
Tensile strength (**a**) and percentage elongation after fracture (**b**) of unremelted and TIG remelting samples under different corrosion periods.

Figure 8 shows the linear regression relationship between corrosion time and the tensile strength, and percentage elongation after fracture of the specimens. The fitted values of a, b, and COD are presented in the table in Figure 8. The tensile strength and elongation at the break of specimens before and after TIG remelting show a linear decrease with the increase in corrosion time. The rate of decline in mechanical properties of TIG-remelted specimens fitted with a straight line is lower than that of unremelted specimens. When the corrosion time is the same, the tensile strength and percentage elongation after fracture of the TIG remelting specimens are higher than those of the unremelted specimens. With the extension of corrosion time, the difference in mechanical properties between the specimens before and after remelting becomes larger. The reason for the occurrence of this phenomenon is that in the unremelted specimen, as corrosion progresses, it is accompanied by the formation of holes (Figure 8), resulting in a reduction in the corresponding cross-sectional area. In addition, during the stretching process, stress concentration occurs near the holes, resulting in a shortened yielding stage and a subsequent decrease in tensile properties. After TIG remelting treatment, the weld toe of the specimen forms a smooth transition, reducing stress concentration and preventing the occurrence of significant corrosion holes on the macroscopic surface of the specimen during the corrosion process. Therefore, the reduction rate of tensile strength and percentage elongation after fracture is lower in TIG remelting specimens than in unremelted specimens.

## 4. Conclusions

TIG remelting improves the geometry of the weld. The stress concentration factor is 1.17 for the unremelting sample, and the remelting sample is 1.06, with a decrease of 9.4% at the weld toe. After TIG remelting of the weld toe, the microstructure of the weld toe is homogenized, and the coarse massive ferrite near the fusion line is transformed into acicular ferrite.

The corrosion resistance of the TIG-remelted sample is better than that of the unremelted sample. The weight loss rate and corrosion rate of the specimen after TIG remelting are lower than those of the unremelted specimen.

The structure of corrosion products is loose at the initial stage of corrosion, and the corrosion products are transformed into Fe_3_O_4_ and Fe_2_O_3_ protective rust layers with dense structure after 480 h of corrosion.

The tensile strength and percentage elongation after fracture of the TIG remelting specimen are higher than those of the unremelted specimen when the corrosion time is the same. With the extension of corrosion time, the tensile strength and percentage elongation of the sample decreased linearly. The decreasing rates of tensile strength of remelted and unremelted specimens were 0.09 and 0.11, respectively, and the decreasing rates of elongation after fracture were 0.0061 and 0.0076, respectively.

## Figures and Tables

**Table 1:** Chemical composition of Q450NQR1 high-strength weathering steel and HTW-55.

Materials	Chemical Composition (wt%)								
	C	Mn	Si	P	S	Cu	Ni	Cr	Fe
Q450NQR1	≤0.12	0.70–1.50	≤0.40	≤0.002	≤0.008	0.25–0.45	0.05–0.40	0.40–0.90	Bal.
HTW-55	≤0.12	≤1.60	≤0.60	≤0.025	≤0.025	-	0.20–0.60	0.30–0.90	Bal.

**Table 2:** Welding Process Parameters.

Steel Plate Number	Welding Process	Voltage (V)	Current (A)	Welding Speed (cm min^−1^)	Shielding Gas Flow Rate (L min^−1^)
Q450NQR1	MAG	26	260	55	18
	TIG	16	170	25	15

**Table 3:** Welding Joint Parameters.

Welding Process	Weld Reinforcement h (mm)	Transition Angle (rad)	Curvature Radius R (mm)
Unremelted(MAG)	3.8	0.63	2
TIG remelting	2.6	0.38	8

**Table 4:** Corrosion weight loss rate and corrosion rate for unremelted and TIG remelting specimens.

Corrosion Time (h)	Unremelted Sample	Mass Loss Rate	Corrosion Rate (g m^−2^ h^−1^)	Remelted Sample	Mass Loss Rate	Corrosion Rate (g m^−2^ h^−1^)
240	A1	2.34	1.63	B1	1.63	1.21
	A2	2.07	1.42	B2	1.76	1.25
	A3	2.28	1.48	B3	1.45	1.23
360	A4	3.36	1.24	B4	2.19	1.02
	A5	3.27	1.38	B5	2.77	1.21
	A6	3.02	1.39	B6	2.36	1.1
480	A7	3.92	1.31	B7	2.95	1.02
	A8	3.39	1.23	B8	2.93	1.08
	A9	3.92	1.27	B9	3.1	1.05
600	A10	3.74	1.03	B10	2.99	1.02
	A11	4.35	1.13	B11	3.46	0.98
	A12	3.54	0.98	B12	3.00	1.03

**Table 5:** Mechanical tensile data for unremelted and TIG remelting specimens.

Corrosion Time (h)	Unremelted Sample	Tensile Strength (MPa)	Percentage Elongation after Fracture (%)	Remelted Sample	Tensile Strength (MPa)	Percentage Elongation after Fracture (%)
240	A1	605	28.4	B1	607	29.1
	A2	605	28.2	B2	609	28.7
	A3	602	28.4	B3	598	28.9
360	A4	600	28	B4	604	27.6
	A5	586	27.6	B5	597	27.6
	A6	588	27.4	B6	602	27.5
480	A7	587	26.8	B7	594	27.2
	A8	572	27.1	B8	587	27.1
	A9	576	27.2	B9	583	27.2
600	A10	564	25.9	B10	577	26.4
	A11	563	25.2	B11	572	26.8
	A12	563	25.4	B12	568	26.5

## Data Availability

All data that support the findings of this study are included within the article.
